# The Role of Urine in Semiochemical Communication between Females and Males of Domestic Dog (*Canis familiaris)* during Estrus

**DOI:** 10.3390/ani10112112

**Published:** 2020-11-13

**Authors:** Martyna Woszczyło, Tadeusz Jezierski, Antoni Szumny, Wojciech Niżański, Michał Dzięcioł

**Affiliations:** 1 Department of Reproduction, Wroclaw University of Environmental and Life Sciences, Plac Grunwaldzki 49, 50-366 Wrocław, Poland; martyna.woszczylo@upwr.edu.pl (M.W.); wojciech.nizanski@upwr.edu.pl (W.N.); 2 Department of Animal Behavior and Welfare, Institute of Genetics and Animal Biotechnology of the Polish Academy of Sciences, Jastrzębiec, 05-552 Magdalenka, Poland; t.jezierski@igbzpan.pl; 3 Department of Chemistry, Wroclaw University of Environmental and Life Sciences, C.K. Norwida 25, 50-375 Wrocław, Poland; antoni.szumny@upwr.edu.pl

**Keywords:** canine reproduction, chemical communication, pheromones, urine, VNO

## Abstract

**Simple Summary:**

Canine reproductive behavior can be easily observed; however, the mechanism of semiochemical signaling in this species is still not well understood. Despite numerous studies, no efficient, artificial canine sex pheromones are available. In most studies of canine semiochemical communication, female urine was believed to be a source of volatile compounds that attract males. We hypothesized that urine is also a source of compounds that are very important in the process of the mating decision but are not so volatile. These compounds are collected by licking urine or the vulva and are transferred into the vomeronasal organ. Such behavior always precedes the male’s mating decision. In two experiments, we assessed the reactions of male dogs in response to air containing odor molecules from estrous females’ urine, from a live female in estrus, and from food, as well as during direct sniffing of urine samples from females in estrus, in anestrus, from male dogs and from humans. It was concluded that urine odor is not used for long-distance semiochemical communication in dogs but rather for close distance signaling.

**Abstract:**

This study aimed to assess the mechanisms of semiochemical signal detection in dogs. In the first experiment, five males were exposed to volatile semiochemicals emitted by a live female in estrus and the female’s urine sample collected during estrus. The odor of canine food and clean air were used as controls. In the second experiment, 25 males could directly sniff and lick the urine samples from females in estrus, from females in anestrus, from males and from humans, placed in a lineup. Sniffing, licking and salivation, as well as keeping dogs at different distances from the source of odor, were recorded in both experiments. Experiment 1 showed that food odor was sniffed by males longer than estrous urine. Volatile semiochemicals from females in estrus evoked interest in males but without visual cues did not cause overt symptoms of sexual arousal. In Experiment 2, the estrous urine evoked interest in males and provoked significantly longer sniffing. Licking accompanied by salivation was observed in all instances only during direct contact with estrous urine. The results suggest a complex character of detection of female reproductive status, in which both volatile and nonvolatile compounds emitted by females and present in female urine are involved.

## 1. Introduction

In 1959, Adolf Butenandt identified bambykol as a sex pheromone in silk moths that attracts the male. Since then, many other sex pheromones in various species have been detected and synthesized [1]. Sexually mature animals respond to pheromone signals from potential mates with endocrine changes and ritualistic behavior patterns [2]. Even though a single compound may act as a pheromone, in most instances, mixtures of compounds are responsible for semiochemical stimulation.

In vertebrates, the vomeronasal organ (VNO) was considered the primary system responsible for pheromone detection [3,4,5], whereas the role of the main olfactory system in reproduction was not studied intensively, assuming that the main olfactory system is responsible for the detection of volatile nonpheromonal odorants in the environment [6]. It is currently believed, however, that the mechanism of pheromone detection may involve the main olfactory system (MOS) and the accessory olfactory system (vomeronasal system (VNS)) or both [1,7,8,9].

The main olfactory system detects airborne scents (volatile chemical components and small airborne peptides) via receptors in the main olfactory epithelium (MOE) even at some distance from their source. On the other hand, the accessory olfactory system detects volatile and nonvolatile molecules that are pumped to the VNO when animals make close nasal contact with them.

It was confirmed in many animal species that males can distinguish the reproductive status of females on the basis of odor [10,11,12,13]. It was believed that pheromones were species-specific; however, there is some evidence of a degree of commonality in estrus odors across species [14]. For example, male rats were able to detect the smell of estrus in feces from mares and vixens [15,16]. A more recent review has shown that a number of pheromones overlap or are cross-reactive between species, especially in invertebrates [17]. Most studies on pheromones have been conducted on insects and on laboratory rodents, with fewer experimental studies dedicated specifically to canine pheromones [1,2].

In domestic dogs, mechanisms of attracting males by a female in estrus were studied and attempts to identify the substance(s) responsible for this phenomenon were undertaken [18,19,20,21,22,23,24,25,26,27]. In most research, urine was considered a main source of attractants, although effective artificial sex pheromones have not yet been definitively identified [20]. In early studies by Goodwin et al. [22], me-4-hydroxybenzoate found in the vaginal secretion of female dogs was proposed as a sex pheromone. In mares, on the other hand, p-cresol in the urine was proposed as one of the components of horse sex pheromone [28]. The results of Dzięcioł et al. [21] indicated differences among samples of urine collected during particular phases of the ovarian cycle, evaluated with the HS-SPME/GC–MS technique. During the period of proestrus and estrus, increased amounts of carbonyl aromatic compounds (acetophenone/hypnone/and benzaldehyde) and methylketones, (2-octanone, 2-pentanone and 3-hexanone) were found. Amounts of sulfide compounds (1-methyltiopropane; 1-methyltiobutane, 1-methylpentane and dimethyl trisulfide) decreased during the period of estrus and increased in diestrus.

Although there have been some studies of canine semiochemical communication in the context of reproduction, sexual communication provoking arousal and the mating decision in dogs is still not fully understood. The aim of this study was to analyze the mechanisms of semiochemical signaling of estrus in female domestic dogs (*Canis familiaris*) and the detection of these signals by adult male dogs. In particular, the role of urine in semiochemical communication between females and males was investigated. 

In our previous study [29], we found that the odor lineup using trained dogs was the most useful method to discriminate estrus odor if multiple testing is required. Not all typical sexual behaviors, however, could be observed with this method, though quantification of the dogs’ responses was considered helpful for future studies on canine sex pheromones. The present study focused on reactions of male dogs to urine odor of females in estrus and the odor of a live female in estrus, when males are exposed to airborne volatile compounds from urine, that are carried across a distance, compared to direct nasal contact to urine samples, including licking.

## 2. Materials and Methods 

### 2.1. Ethical Statement

The research was conducted in accordance with the regulations on animal experimentation and guidelines for the use of animals in research. The 2nd Local Commission for Experiments with the Use of Laboratory Animals, Wrocław, Poland issued a statement approving the experimental protocol (Permission No. 054/2019).

### 2.2. Animals

A total of 25 male dogs were used in this study: five were beagles from the Clinic of Animal Reproduction experimental kennel, and the remaining 20 were of different breeds, made available by local breeders for this research. All animals used in the experiment were clinically examined, and all urine sample donors were examined for kidney metabolic disorders and lower urinary tract diseases. Only healthy animals were used for the experiments. 

### 2.3. Collection of Urine Samples

Urine samples were collected by natural urination from 10 female patients in estrus of the Clinic of Animal Reproduction. These samples were stored at −20 °C for use in the evaluation of semiochemical signals. Urine samples were collected by natural urination and stored at −20 °C. Male and female urine samples were acquired from individuals foreign to the dogs used for the trials. 

Additionally, for comparison, human urine samples were collected from two men and one woman.

### 2.4. Estrus Detection Methods 

The exact stage of the ovarian cycle was determined by clinical examination and laboratory tests (vaginal cytology and measurement of progesterone concentrations) [21,30,31]. Progesterone concentration in peripheral blood was determined by enzyme-linked fluorescence assay (ELFA; mini VIDAS^®^ Biomerieux, Craponne, France) [32]. To confirm female attractiveness to the males, direct contact was allowed and sexual behavior (signs of arousal, sniffing, licking, salivation, mating attempts) was evaluated. For this contact, other males than those used in the present experiments were employed. 

### 2.5. Experimental Design

#### 2.5.1. Experiment 1

Experiment 1 aimed at evaluating the reactions of the male dogs to the volatile signal characteristic of estrus. Five adult, intact Beagle males, aged 4–6 years, were exposed to different odors, on the assumption that only volatile compounds would be perceived by the dogs. All the males had previous contact with other females in estrus and were experienced estrus detection. 

##### Odor Sample Presentation to the Dogs

Four trials were conducted within Experiment 1 with different kinds of volatile attractants: Trial 1—with clean air and no urine as the attractant; Trial 2—with a urine sample of 10 mL, collected from females in estrus; Trial 3—with a live female in estrus; and Trial 4—with canine food as the attractant. 

**Figure 1:**
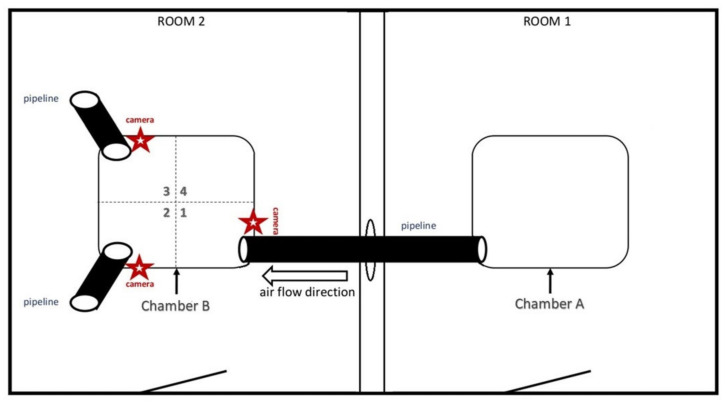
Visual scheme of Experiment 1.

For this experiment, a special device was designed (Figure 1). Steel chambers, A and B, each 1.5 m long, 1.5 m wide and 1.2 m high, with firm walls, were placed in each of two separate and well-ventilated rooms connected only by a culvert, 5 cm in diameter, through the walls. Both chambers were connected by a pipeline, 5 cm in diameter, through which unidirectional air movement was directed using a fan. Through the pipeline, air from Chamber A containing the source of odor was pumped into Chamber B, where an adult male dog was placed. Additionally, two pipelines pumping clean air from another location, uncontaminated by any odor, were placed in two opposite corners of Chamber B (Figure 1). The air was pumped simultaneously with the same intensity through all three pipelines.

In Trial 2, a urine sample of 10 mL, collected from females in estrus, was placed in Chamber A in direct contact with the air pipeline at room temperature.

Before the main experiment, several blank trials were conducted to habituate the dogs to the laboratory environment, including putting them in a chamber and getting them accustomed to the gentle sound of the fan pumping the air. The habituation of males was conducted before the habituation of females, applying only clean air pumped through all the pipelines. Between particular trials performed at intervals of a few days, both rooms were intensively ventilated. During initial trials as well as during the main experiment, the dogs remained in eye contact with the lab attendant, known to the animals, to reduce the risk of stressful reactions by the animals.

**Figure 2:**
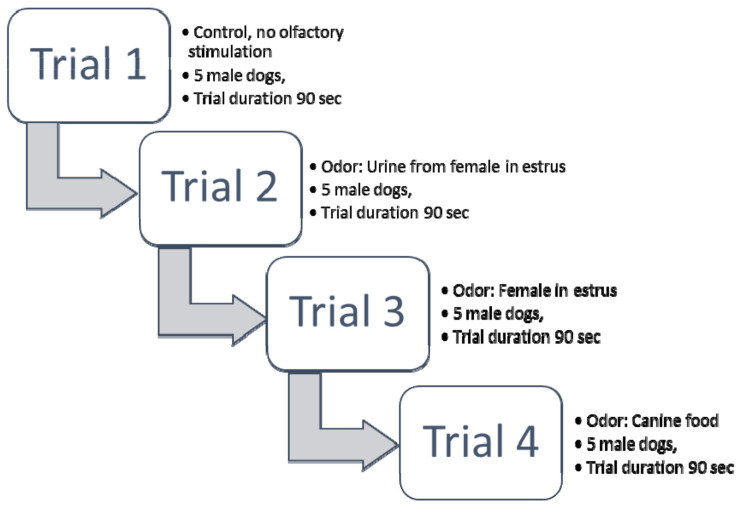
Scheme of Experiment 1.

Trials were conducted at intervals of a few days in the order shown in Figure 2. Trial 4 confirmed the functionality of the designed device and the usefulness of the experimental model. Trial 4 with food was conducted last to prevent triggering a learned reaction from the dogs, which might expect interesting odors flowing from the pipelines, thereby biasing the results of the trials with other odors.

In Trial 1, the male was placed in Chamber B, whereas Chamber A was empty. During this trial, clean air from Chamber A was pumped into Chamber B, and clean air from another location was pumped into Chamber B as a blank control.

During Trials 2, 3 and 4, the male was again placed in Chamber B and, in Chamber A, an estrus urine sample (Trial 2), live female in estrus (Trial 3) or canine food (Trial 4) were placed. In Trial 3, no visual and/or direct olfactory contact (except via air pumped through the pipeline) was possible between the male and female placed in Chambers A and B in separate rooms (Figure 1). To avoid the impact of unexpected acoustic stimuli, gentle music was played in the room with the male. Different persons were in charge of transporting male and female dogs to the experimental site to avoid contamination of the attendant’s clothes with estrus or food odors. 

Between particular trials in Experiment 1, the plastic pipelines were exchanged to avoid contamination with the odor from the previous trial. In all instances, trials were repeated three times.

##### Behavioral Analyses

During each trial, the behavior of the males was recorded using three video cameras (Figure 1). Each trial took 90 s. The behavioral repertoire was evaluated with special attention paid to attempts at sniffing, directly sniffing the outlet of a pipeline, licking, and to the frequency of approaching the outlets of a particular pipeline. For a detailed analysis of the dogs’ behavior in Chamber B, the floor of the chamber was divided into 4 sectors—1, 2, 3 and 4—with Sector 1 being the closest to the pipeline outlet. The Observer XT software (Noldus, Wageningen, The Netherlands) was used for the analysis of the dogs’ behavior. After the end of Trial 3, on the same day, close contact between the male dogs and the female in estrus was allowed to confirm the attractiveness of the female and the males’ ability to detect estrus. 

To avoid bias, the person who analyzed the video records of the dogs’ behavior was blind to the particular trial they evaluated.

#### 2.5.2. Experiment 2

The aim of Experiment 2 was to evaluate the reaction of the male dogs to various samples of urine collected from females in different phases of the reproductive cycle (estrus vs. anestrus), as well as from male dogs and from humans. 

Twenty-five adult, intact males of different breeds with a mean age of 4.4 years (±1.92) were used for Experiment 2. All dogs had had previous contact with females in estrus and demonstrated signs of sexual arousal, confirming their ability to detect estrus in females. 

##### Odor Sample Presentation to the Dogs

**Figure 3:**
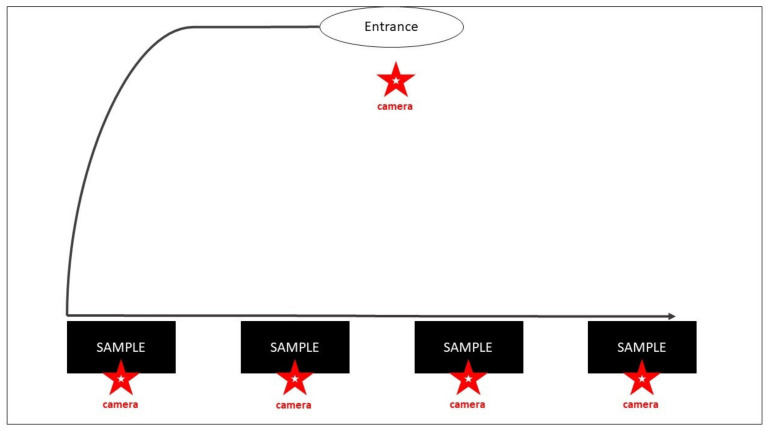
Scheme of Experiment 2.

In the empty experimental room (35 m^2^ in area), four identical plastic boxes (10 × 20 cm) were placed on the floor in a lineup (Figure 3). On the upper, transparent surface of each box, a different sample of 0.5 mL urine was applied: Sample A—urine from females in anestrus; Sample B—urine from a male; Sample C—human urine; and Sample D—urine from a female in estrus. Samples A–D, in each trial, were placed in random order, and the dog handler was blind to the position of particular samples.

##### Behavioral Analyses

During the trials, the male dogs were gently led on a leash along the lineup of boxes. The behavior of the dogs was recorded by one main video camera. Additionally, small cameras were placed in all the plastic boxes to acquire detailed records of the sniffing behavior of the male dogs during contact with the particular samples (sample examination time, occurrence and duration of sniffing, licking, salivation, and so forth). The results were evaluated using the Observer XT software. The person who evaluated the video recordings was blind to the order of the samples in the lineup.

### 2.6. Statistical Methods

Data were evaluated using the one-way ANOVA test and the post hoc Fisher Least Significant Difference (LSD) test, with a significance level of *p* < 0.05 (STATISTICA Data Analysis Software System, version 13, TIBCO Software Inc., 2017). For differences in the number of dogs performing licking and salivation, the chi-square test was used.

## 3. Results

### 3.1. Experiment 1

**Figure 4:**
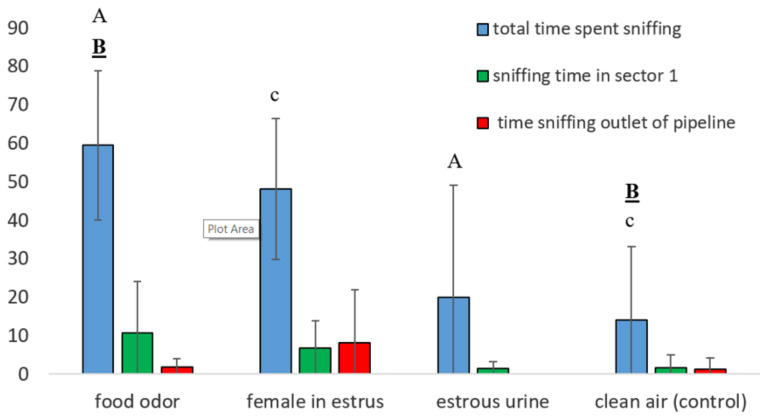
Sniffing time in seconds (mean ± s.d.) in particular places of Chamber B in Experiment 1. The same letters over bars denote significant differences. Bold underlined capitals, *p* < 0.001; capitals, *p* < 0.01; lowercase, *p* < 0.05.

In Experiment 1, the dogs demonstrated more interest in food odor compared to the other odors, taking into account the total sniffing time (Figure 4), which confirmed the proper functioning of the device. The Least Significant Difference (LSD) post hoc tests showed that total time sniffing the food odor was longer than sniffing the estrous urine (*p* < 0.01) and clean air (*p* < 0.001, Figure 4). The odor of a female in estrus was sniffed longer than clean air (*p* < 0.05, Figure 4). When particular sectors within Chamber B were taken into consideration, we found that in the trials with food and with the female in estrus, the dogs spent more time sniffing in Sector 1, closest to the outlet of the pipeline through which the odor was pumped, but the difference was not statistically significant (Figure 4). No significant differences were found between any two trials in the duration of other behaviors such as standing, sitting or lying down.

Behaviors typical of sexual arousal and VNO stimulation, such as excessive salivation and licking of the pipeline outlet, were not observed in any of the trials. The dogs did not demonstrate searching behavior to find other sources of odor besides the pipeline outlet. 

**Figure 5:**
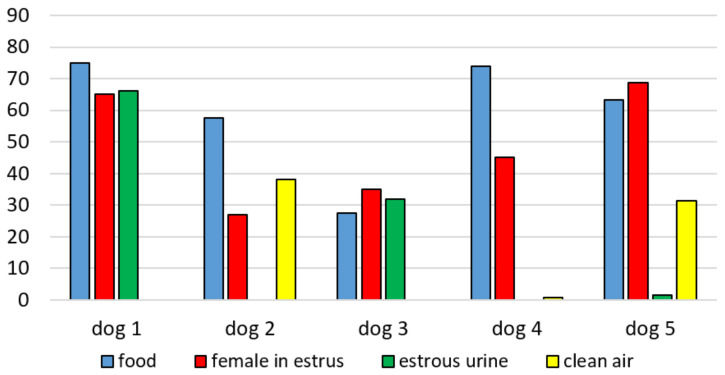
Percent of total sniffing time of different odors in individual dogs in Experiment 1.

Since only five males were available for Experiment 1 and because of variability between them, individual differences between dogs were analyzed (Figure 5). All dogs were interested in food odor and the odor of a female in estrus. Only two dogs (Dog 2 and Dog 4) sniffed food odor longer than the odor of a female in estrus (Figure 5), and only two other dogs (Dog 1 and Dog 3) were interested in the odor of estrous urine. On the other hand, Dogs 2 and dog 5 were, to some extent, interested in the odor of clean air (Figure 5).

### 3.2. Experiment 2

**Figure 6:**
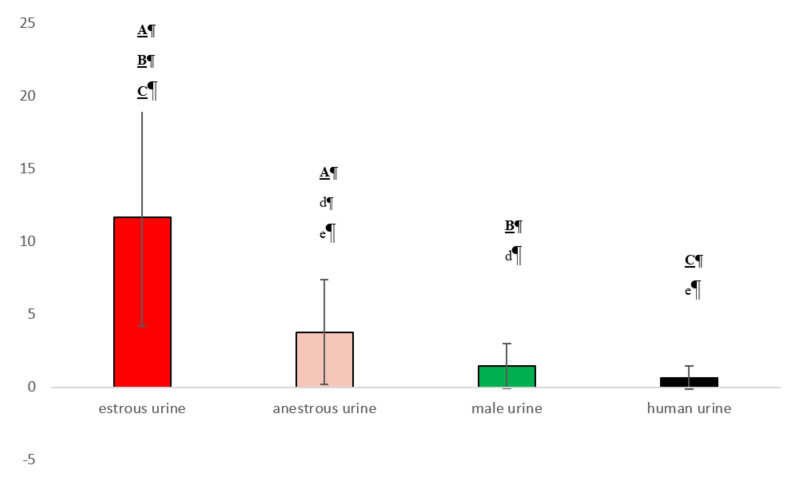
Total duration of sniffing in Experiment 2 (seconds, mean ± s.d.). The same letters over bars denote significant differences. Bold underlined capitals, *p* < 0.001; capitals, *p* < 0.01; lowercase, *p* < 0.05.

In Experiment 2, the dogs mostly avoided or did not sniff the samples containing human urine. Thus, the mean sniffing time of the human urine was the shortest (Figure 6). Samples containing urine of females in anestrus were investigated longer than human and male urine samples (*p* < 0.05, Figure 6). In two out of the twenty five dogs, attempts to lick the anestrous samples were observed. The greatest interest and the longest sniffing time (*p* < 0.001, Figure 6) were observed for the urine samples collected from a female in estrus.

All 25 male dogs demonstrated licking behavior for the estrous urine sample. Two dogs licked the anestrous sample, and one dog licked the male urine sample. The difference in the number of dogs that licked the estrous sample and those that licked the anestrous or male urine samples was significant at *p* < 0.001 (chi-square test). Twelve dogs out of 25 showed a specific facial muscle reaction in contact with estrous urine samples, and one dog showed this behavior in contact with anestrous urine (*p* < 0.01). Excessive salivation was observed in nine dogs, only during contact with estrous urine (*p* < 0.01).

## 4. Discussion

In many animal species such as guinea pigs [33], rats [34], mice [35], hamsters [36] and ferrets [37], both sexes show a preference for investigating pheromonal signals from the opposite sex. According to Hurst [38] and Martinez-Garcia et al. [39], this preference to investigate the pheromonal signal of the opposite sex is apparent when direct nasal contact with the pheromone is possible, which leads to the activation of the VNO receptors. This may explain why, in Experiment 1, the male dogs did not show more interest for the airborne odor of estrous urine than for clean air, but in Experiment 2, the dogs showed more interest for samples of estrous urine than for anestrous or male urine, because there was the possibility of direct nasal contact with the urine samples, even the opportunity to lick the urine.

Results obtained in Experiment 1 showed that the dogs were interested in the volatile airborne odors coming from the chamber where a live female in estrus was placed. The scent of this female attracted the males, stimulated them to sniff and induced some kind of minor arousal, as expressed by their spending more time standing and exploring the area near the outlet of the pipeline. This scent, however, did not induce typical signs of specific sexual arousal such as licking the source of the odor (in this instance, the outlet of the pipeline) or excessive salivation, freezing and intense seeking of the female. This could be an indication that volatile semiochemical signals were detected, but they alone were not sufficient to induce a full behavioral response. This observation seems consistent with clinical practice, showing that during attempts to stimulate a male dog for semen collection in the absence of a female in estrus, the presentation of estrous odor on the swabs results only in very weak sexual arousal in the male. These results seem consistent with the reports of researchers who had studied similar behaviors in different species [8].

The analysis of individual differences between dogs showed that their individual preference for airborne food odor vs. female in estrus or estrous urine odor is variable. This may confirm that the male dogs receive these odors from a distance, but they do not differentiate the odors by importance. 

In Experiment 1, the dogs showed no interest in the odor of estrous urine coming out from the pipeline. The duration of sniffing of the estrous urine was similar to the duration of sniffing of the clean air. The present results are consistent with previously published results, showing that in the field test, male dogs can detect a sample of estrous urine with a surprisingly small degree of accuracy and rather by accident [27]. In the study by Jezierski et al. [29], samples of estrous urine placed in the vulval region of an artificial dog model elicited a strong interest and signs of sexual arousal in male dogs. These different reactions could be explained by the fact that the dogs were attracted to the dog model not by a semiochemical signal contained in the urine but first visually, since the object resembled a dog. Subsequent stimulation was elicited by the simultaneous action of a visual stimulus, i.e., the artificial dog model, and olfactory stimulus, i.e., the odor of the urine sample placed in the model [29]. Interestingly the simultaneous visual and olfactory stimulation by the artificial dog model significantly decreased in consecutive trials [29]. 

The results of Experiment 1 show that volatile semiochemicals alone produced by a female dog in estrus, without respective visual stimuli, are not sufficient to induce a full sexual response in male dogs. It could be supposed that urine alone is not a source of volatile semiochemicals that can be easily detected by a male dog or that can attract a male dog from a distance. Experiment 1, however, shows that male dogs are interested in the odor of a female in estrus even if the female is not visible. It could be supposed that some volatile compounds responsible for sexual stimulation are present, not in urine, but emitted by glands on the body of a female, e.g., on the paws or in the vulval region. The molecules in the odor transported through the pipeline in Experiment 1 had to be very volatile, e.g., the molecules of the food odor, to be perceived by the dogs. The results of Experiment 2, where the dogs were exposed to urine odors of different kinds from a very close distance and could both sniff and lick the urine, seem to confirm the importance of nonvolatile semiochemicals present in urine, since the male dogs showed more interest for estrous urine than for the other kinds of urine. Thus, urine may be regarded as a source of nonvolatile chemical messages in the context of reproductive behavior, even though the mechanism of transmission seems not to be connected with the presence of volatile compounds [40,41]. Substances that are found in urine samples from females in estrus may stimulate male dogs to seek closer and longer contact, accompanied by sniffing and licking. This observation is consistent with the reports of Doty and Dunbar [18], who found that sexually experienced males spent more time investigating estrous than diestrous female urine. We observed not only a longer time of sniffing of estrous urine in Experiment 2 but also a characteristic licking and introduction of the licked urine into the VNO by tongue flicking, a process known as flehmen [42]. Thus, the less volatile or nonvolatile compounds in the urine could be introduced into the VNO for better examination and assessment of the “semiochemical status” of the urinating female. A similar mechanism can be clearly observed in both, Asian (*Elephas maximus*) and African elephants (*Loxodonta africana*), which used their trunk to collect from the ground urine samples of the females being in the preovulatory phase of the ovarian cycle [43,44].

Behavioral reactions of a male towards a female in estrus are not simple reflexes but rather a complex mechanism consisting of several phases, including approaching, sniffing and licking. Licking can be observed when a male tries to pick up small amounts of a female’s urine from the spot where the female had just urinated, but male dogs also intensely lick a female’s vulva before attempting to mate the female. It seems that the licking of urine and the vulva enables the assessment of a female’s receptivity. Previously published results, as well as the present study, suggest that only a full range of stimuli, including volatile and nonvolatile compounds perceived by both the main and accessory olfactory systems and acting together with visual stimuli, can elicit the full range of typical sexual behavior and strong arousal [29].

It was found that several volatile chemicals like androgenic pig steroid (androstenone) act as pheromones, influencing behavior and neuroendocrine function after they have been received by receptor neurons in the main olfactory epithelium (MOE) [45,46,47,48,49]. Slotnick et al. [50] suggested that the interaction between the main olfactory system and the VNO consists of a pumping mechanism of the main olfactory system that contributes to the delivery of pheromones to the VNO.

We could not find in the literature studies identifying specific canine vomeronasal receptors. Such studies were conducted primarily on mice. For example, Haga-Yamanaka et al. [51] identified mouse vomeronasal receptors responsible for detecting female pheromones. The authors found that a sub-group of V1re clade members recognizes gender-identifying cues in female urine. However, multiple members of the V1rj clade are responsible for the recognition of urinary estrus signals and sulfated estrogen (SE). Haga-Yamanaka et al. [51] also found that the same cue activates multiple homologous receptors, which suggests redundancy in encoding female pheromone cues. Neither gender-specific cues nor SEs alone were sufficient to promote courtship behavior in male mice, whereas robust courtship behavior was induced when the two cues were applied together. Thus, the integrated action of different female cues is required in pheromone-triggered mating behavior.

## 5. Conclusions

This study indicates that the odor of the urine of a female dog in estrus, placed at a distance from male dogs and without any visual stimuli or the possibility of direct contact, i.e., licking, does not evoke sexual interest or stimulation of male dogs. Volatile constituents of the body odor of live females in estrus evoke interest in males, but they alone do not stimulate overt sexual behavior. Direct contact with estrous urine stimulates males for close nasal contact, accompanied by the licking of the urine. This may be an indication that the estrous urine contains semiochemically active, nonvolatile compounds that need to be identified in chemical analyses.
